# Chromosomal Rearrangements and Satellite DNAs: Extensive Chromosome Reshuffling and the Evolution of Neo-Sex Chromosomes in the Genus *Pyrrhulina* (Teleostei; Characiformes)

**DOI:** 10.3390/ijms241713654

**Published:** 2023-09-04

**Authors:** Renata Luiza Rosa de Moraes, Francisco de Menezes Cavalcante Sassi, Jhon Alex Dziechciarz Vidal, Caio Augusto Gomes Goes, Rodrigo Zeni dos Santos, José Henrique Forte Stornioli, Fábio Porto-Foresti, Thomas Liehr, Ricardo Utsunomia, Marcelo de Bello Cioffi

**Affiliations:** 1 Departamento de Genética e Evolução, Universidade Federal de São Carlos, São Carlos 13565-905, SP, Brazil; rlrdm@hotmail.com (R.L.R.d.M.); francisco.sassi@hotmail.com (F.d.M.C.S.); jhonalex279@gmail.com (J.A.D.V.);; 2 Institute of Human Genetics, University Hospital Jena, 07747 Jena, Germany; 3 Faculdade de Ciências, UNESP, Bauru 17033-36, SP, Brazil; caioaggoes@gmail.com (C.A.G.G.); rodrigo.zeni@unesp.br (R.Z.d.S.); fp.foresti@unesp.br (F.P.-F.); ricardo.utsunomia@unesp.br (R.U.); 4 Institute of Biological Sciences and Health, Universidade Federal Rural do Rio de Janeiro, Seropédica 23890-000, RJ, Brazil; jose.henrique@unesp.br

**Keywords:** satDNAs, lebiasinidae, sex chromosomes, karyotipic reduction

## Abstract

Chromosomal rearrangements play a significant role in the evolution of fish genomes, being important forces in the rise of multiple sex chromosomes and in speciation events. Repetitive DNAs constitute a major component of the genome and are frequently found in heterochromatic regions, where satellite DNA sequences (satDNAs) usually represent their main components. In this work, we investigated the association of satDNAs with chromosome-shuffling events, as well as their potential relevance in both sex and karyotype evolution, using the well-known *Pyrrhulina* fish model. *Pyrrhulina* species have a conserved karyotype dominated by acrocentric chromosomes present in all examined species up to date. However, two species, namely *P. marilynae* and *P. semifasciata,* stand out for exhibiting unique traits that distinguish them from others in this group. The first shows a reduced diploid number (with 2*n* = 32), while the latter has a well-differentiated multiple X_1_X_2_Y sex chromosome system. In addition to isolating and characterizing the full collection of satDNAs (satellitomes) of both species, we also in situ mapped these sequences in the chromosomes of both species. Moreover, the satDNAs that displayed signals on the sex chromosomes of *P. semifasciata* were also mapped in some phylogenetically related species to estimate their potential accumulation on proto-sex chromosomes. Thus, a large collection of satDNAs for both species, with several classes being shared between them, was characterized for the first time. In addition, the possible involvement of these satellites in the karyotype evolution of *P. marilynae* and *P. semifasciata*, especially sex-chromosome formation and karyotype reduction in *P. marilynae,* could be shown.

## 1. Introduction

Fishes are an incredibly diverse group with many chromosomal variations, including polyploidy, supernumerary chromosomes, distinct sex chromosome systems, and polymorphisms [1]. In fact, most cases of postzygotic isolation are caused by genetic incompatibilities, among which chromosomal rearrangements play a fundamental role [2,3,4]. Chromosomal changes have the potential to limit introgression, thus facilitating the origin and maintenance of reproductive isolation through recombination suppression [5,6]. However, one of the most interesting evolutionary events refers to the emergence of neo-sex systems, when multiple sex chromosomes arise because of rearrangements between an autosome and a sex chromosome. This evolutionary step, also known as sex chromosome turnover, has the potential to suppress recombination next to breakpoints, creating new linkage groups between genes from distinct chromosomes, increasing the number of sex-linked genes, and accelerating the accumulation of genetic incompatibilities between populations [7].

The impact of chromosomal rearrangements in fish karyotype evolution has been studied primarily from a cytogenetic point of view, with a particular emphasis on the chromosomal mapping of repetitive DNA sequences. The latter has proven to be a valuable source of information on the role of such sequences in genome organization and evolution [8,9]. Satellite DNAs (satDNAs) are one of the most common repeated sequences, forming extensive arrays of largely similar repeating units (monomers) that make up a significant percentage of genomes (reviewed in [10]). Recently, given the integration of cytogenetics with high-throughput sequencing data from next-generation sequencing methods (NGS), the whole collection of different satDNA families (satellitome) of several species has been characterized, providing insights into several evolutionary issues, such as karyotype evolution, genome diversity, and phylogenetic relationships [11,12,13,14,15,16,17,18,19,20]. These satellites (satDNAs) are also thought to play a role in the evolution and structure of sex chromosomes, as well as chromosome-based speciation [13,14,21,22,23,24,25].

*Pyrrhulina* Valenciennes 1846 (Characiformes, Lebiasinidae) is the most diverse genus of the fish subfamily Pyrrhulininae, with 19 valid species [26]. Many species remain unexplored due to their small sizes and, thus, difficult sampling; consequently, the genus presents unsolved taxonomic issues [27]. Recent research, however, has led to a better understanding of the evolution of Lebiasinidae species, including several *Pyrrhulina* ones, particularly from a cytogenetic and molecular genetics standpoint [28,29,30,31,32,33,34,35,36]. In general, about half (i.e., nine out of the 19) *Pyrrhulina* species have been cytogenetically documented, demonstrating a quite conserved diploid chromosome number, ranging from 40 to 42 chromosomes with karyotypes predominantly formed by acrocentric chromosomes [28,29,30,37]. Aside from distinctive karyotypes and diploid numbers, genomic content comparisons among all analyzed species reveal a significant degree of similarity between their genomes, with most of the variations related to their repetitive content [30]. Most species have multiple 5S rDNA and 18S rDNA sites, with some species having a syntenic arrangement of these rDNAs [28,29,30]. Among all *Pyrrhulina* species, *P. marilynae* and *P. semifasciata* stand out for exhibiting characteristics that distinguish them from other species in the genus. In the first case, *P. marilynae* shows a significant karyotypic reduction, presenting 2*n* = 32 chromosomes with four metacentric pairs not observed in other species, presumably due to secondary fusions [30]. *P. semifasciata*, on the other hand, contains the sole morphologically differentiated sex chromosome system found in the genus, i.e., the multiple X_1_X_1_X_2_X_2_/X_1_X_2_Y system [29].

In this study, we selected those two *Pyrrhulina* species that underwent substantial, cytogenetically visible chromosomal rearrangements to examine the involvement of satDNAs in these chromosome-shuffling events and their putative role in both sex and karyotype evolution. Apart from performing a comprehensive analysis of their satellitomes, the satDNAs located on *P. semifasciata*’s sex chromosomes were also mapped in two phylogenetically related species (*P. brevis* e *P. obermulleri*) to check their possible accumulation on proto-sex chromosomes. 

## 2. Results

### 2.1. SatDNA Content of P. marilynae and P. semifasciata

We applied the satMiner pipeline using short-read libraries of *P. semifasciata* (female) and *P. marilynae* (for more detailed information, see the material and methods section). After three iterations of each of the satMiner protocols, we found 70 and 71 satDNA families for *P. marilynae* (Pma) and *P. semifasciata* (Pse), respectively. The repeat unit lengths ranged from 23 to 4663 bp, with a median of 443.5 bp for *P. marilynae*, and from 6 to 2510 bp, with a median of 39 bp in *P. semifasciata*. In *P. marilynae*, the A + T content of satDNAs ranged from 39.2 to 71.8% with a mean of 60%, whereas in P. semifasciata, it ranged from 39.2 to 78.5% with a mean of 60%. In total, 64 and 77 satDNAs in P. marilynae and P. semifasciata, respectively, had an A + T content of more than 50%. Long satDNAs (>100 bp sensu [12]) were predominant in both satellitomes, with 39 and 44 satDNA families in *P. marilynae* and *P. semifasciata,* respectively. The complete results for each satellitome are described in Tables S1 and S2. Sequences are available on the NCBI-Genbank, under the accession numbers OR094701-OR094771 (*P. semifasciata*) and OR094772-OR094841 (*P. marilynae*).

### 2.2. Chromosomal Distribution of PmaSatDNA in P. marilynae

**Figure 1:**
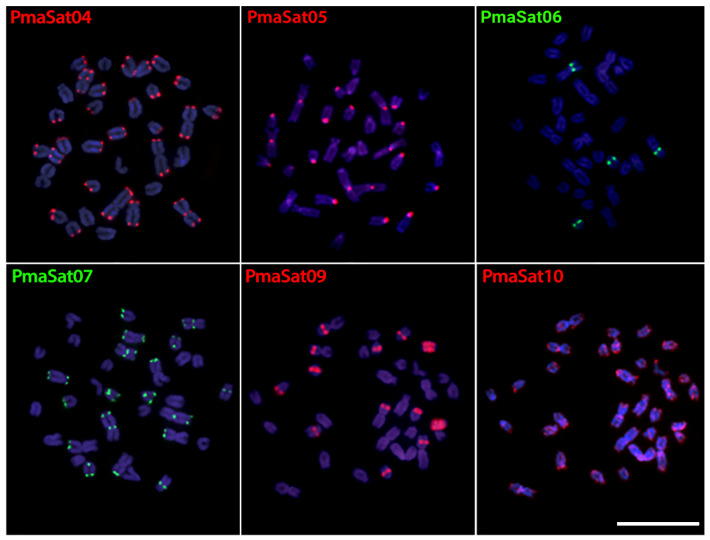
Metaphase plates of *Pyrrhulina marilynae* highlighting the chromosomal location PmaSatDNAs. The satDNA family names are indicated on the left top, in red (Atto550-labeled) or green (Atto488-labeled). Scale bar = 10 µm.

To examine the chromosomal location and distribution of the PmaSatDNAs, we used both female and male mitotic metaphase plates of *P. marylinae* in our two-color fluorescence in situ hybridization (FISH) experiments. Within 10 successfully amplified satDNAs families, six of them produced visible FISH signals, yielding the same result in both sexes (Figure 1). PmaSat04, PmaSat07, and PmaSat10 were mostly found in the telomeric and centromeric regions of most chromosomes, with the presence of bitelomeric signals for PmaSat04 and PmaSat10 (Figure 1). In addition, PmaSat06, PmaSat07, PmaSat09, and PmaSat10 were observed in the pericentromeric regions of some chromosomes (Figure 1). The satDNAs PmaSat02, PmaSat03, and PmaSat08 did not produce any FISH signal.

### 2.3. Chromosomal Distribution of PseSatDNA in P. semifasciata

**Figure 2:**
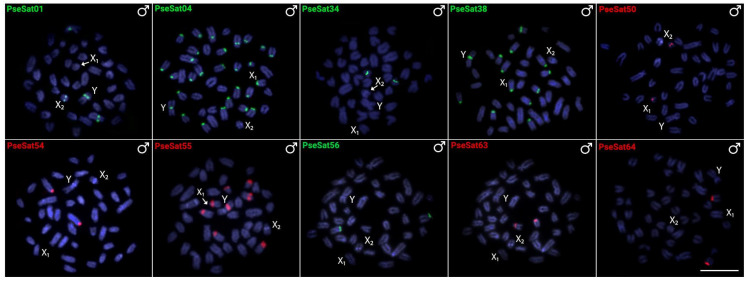
Male metaphase chromosomes of *Pyrrhulina semifasciata* after FISH with 10 PseSatDNAs. The satDNA family names are indicated in the top left corner in red (Atto550-labeled) or green (Atto488-labeled). The sex chromosomes X_1_, X_2_, and Y are indicated. Scale bar = 10 µm.

To examine the chromosomal location and distribution of PseSatDNAs, we used both female and male mitotic metaphase plates of *P. semifasciata* in the same two-color FISH sets as before for *P. marilynae*. Within the 16 successfully amplified satDNA families, ten produced visible FISH signals, yielding the same result in both sexes, except the ones located on the sex chromosomes. Most of the analyzed PseSatDNAs were found in the centromeric and pericentromeric regions of the autosomal chromosomes (Figure 2). PseSat01, PseSat04, PseSat38, and PseSat55 hybridized in the autosomes and sex chromosomes of *P. semifasciata* (Figure 2). PseSat01, the most abundant satellite DNA, was located on two autosomes, the X_2_ and the Y chromosome (Figure 2 and Figure S1). The sequences PseSat06, PseSat32, PseSat39, PseSat57, PseSat61, and PseSat67 did not produce any visible FISH signals. 

**Figure 3:**
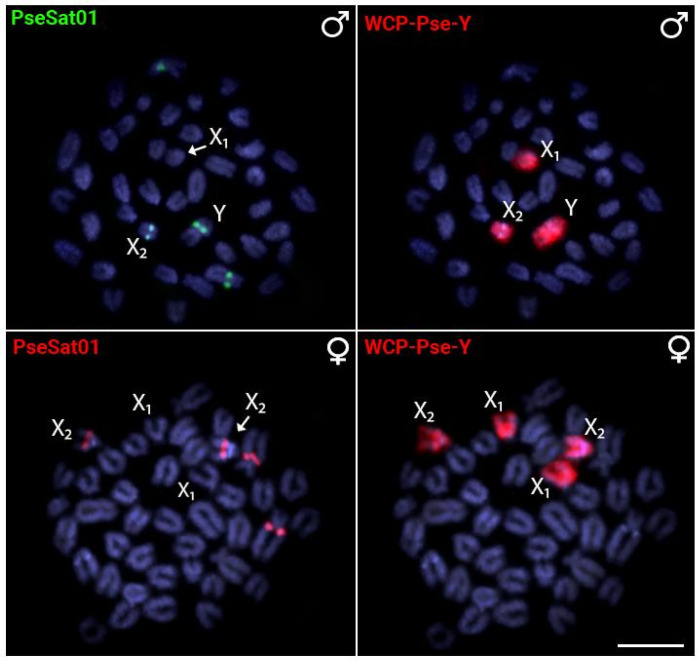
Male and female metaphase plates of *Pyrrhulina semifasciata* showing that the hybridization pattern of PseSat01 (first column) is coincident with the X_2_ and Y sex chromosomes, as indicated by the whole-chromosome painting with the PSEMI-Y probe (second column), which is derived from the microdissection of the Y chromosome. Scale bar = 10 µm.

In all experiments, a second FISH experiment using PseSat01 and/or the Y-specific PSEMI-Y probe was carried out to accurately identify the X_1_, X_2,_ and Y sex chromosomes (Figure 3).

### 2.4. Chromosomal Distribution of PseSatDNA in Other Pyrrhulina Species

**Figure 4:**
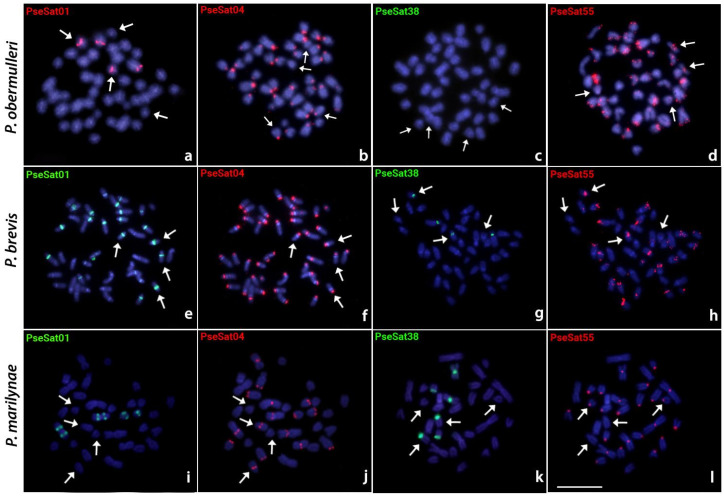
Metaphase plates of *Pyrrhulina obermulleri* (**a**–**d**), *P. brevis* (**e**–**h**), and *P. marilynae* (**i**–**l**) highlighting the chromosomal location of PseSatDNAs that were mapped in the sex chromosomes of *P. semifasciata*. The satDNA family names are indicated in the upper left in red (if labeled with Atto550-dUTP) or green (if labeled with Atto488-dUTP). The arrows indicate the proto-sex chromosomes. Scale bar = 10 µm.

**Figure 5:**
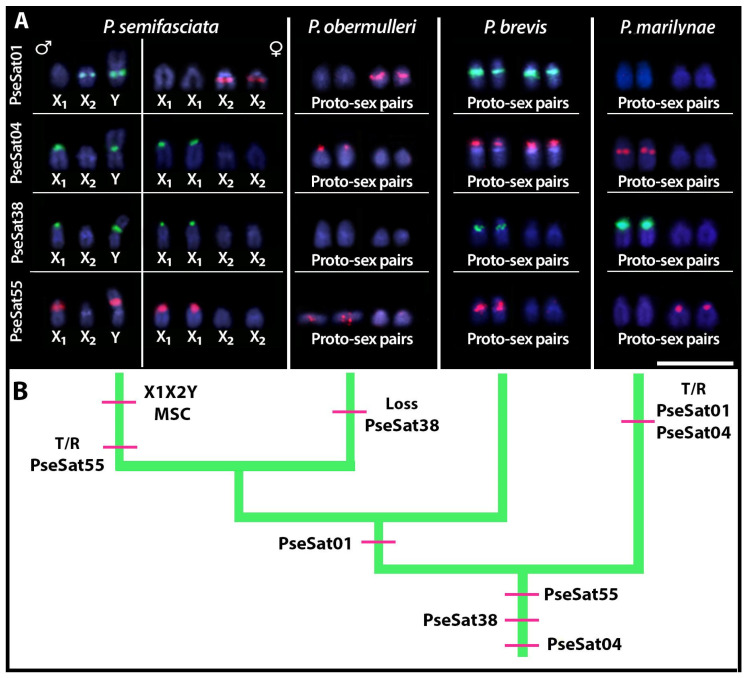
(**A**) *Pyrrhulina semifasciata* male (first column) and female (second column) sex chromosomes highlighting the hybridization pattern of the four PseSatDNAs that produced visible signals, with each line matching to a satellite sequence indicated on the left. The third, fourth, and fifth columns show the hybridization patterns of those satellites in the *P. obermulleri*, *P. brevis*, and *P. marilynae* chromosomes, respectively. Scale bar = 10 µm. (**B**) Phylogenetic relationships of *Pyrrhulina* species (green lines) based on [38] plotted with main events of origin, loss, and T/R (transposition or recombination) of PseSatDNAs, and origin of the multiple sex chromosome system (MSC) (pink lines).

All PseSatDNAs located on the sex chromosomes of *P. semifasciata* (i.e., PseSat01, PseSat04, PseSat38, and PseSat55) were also hybridized against *P. obermulleri*, *P. brevis*, and *P. marilyanae* metaphase chromosomes. All these satDNAs delivered evaluable results in all species (Figure 4), except for PseSat38, which was not visible in the chromosomes of *P. obermulleri* (Figure 4c). PseSat04 hybridized in the centromeric region of almost all chromosomes in *P. obermulleri* and *P. brevis* (Figure 4b,f), whereas in *P. marilynae*, visible signals were seen in the centromeric, pericentromeric, or telomeric regions on most chromosomes (Figure 4j). PseSat01, on the other hand, was present in only two chromosome pairs in *P. obermulleri* and *P. marilynae* (Figure 4a,i) and in nearly all chromosomes in *P. brevis* (Figure 4e). PseSat55 was found in the centromeric and telocentromeric regions of most *P. obermulleri* and *P. brevis* chromosomes (Figure 4d,h). In *P. marilynae*, on the other hand, it was mapped exclusively in the centromeric region of most chromosomes (Figure 4l). Except for PseSat38, which was not present in *P. obermulleri*, and PseSat01, which did not exhibit visible signals in *P. marilynae* proto-sex pairs, all three species had at least one pair of putative proto-sex pairs that exhibited positive signals for each of the selected PseSatDNAs (Figure 5). Again, in all slides, a second FISH experiment with the Y-specific PSEMI-Y probe was carried out to accurately identify the proto-sex chromosomes.

### 2.5. Minimum Spanning Trees: MSTs

**Figure 6:**
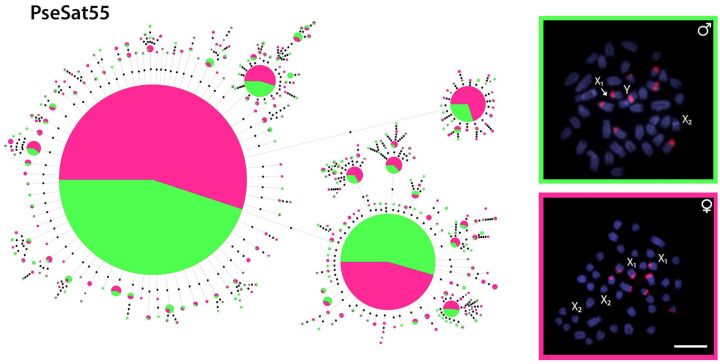
Linear MSTs of PseSat55 obtained from reads of females (pink) and males (green). The diameter of the circle is proportional to the abundance of the haplotype. Black circles represent 01 base of divergence between haplotypes, and the black triangles represent 05 bases of divergence. The in situ mapping in male and female metaphase plates is highlighted in boxes. Bar = 5 µm.

We have chosen PseSat55 to generate minimum spanning trees (MST). The other satDNAs clustered in *P. semifasciata*’s sex chromosomes contain more than 150 bp, making it impossible to create an MST. In addition to the identical locations of the X_1_ and Y chromosomes, PseSat55 is found in the pericentromeric regions of six autosomes in both males and females (Figure 6). Even among the less common haplotypes, this sharing is observed, with only a few sequences being exclusive, mostly in females. The MST results show that PseSat55 is homogeneous in both sexes, with just a few unique haplotypes, suggesting a certain degree of recombination between the X and Y chromosomes.

## 3. Discussion

### 3.1. General Features P. marilynae and P. semifasciata Satellitomes

Here, we show that a significant proportion of satDNA families (38 satDNAs) are shared between the satellitomes of *P. marilynae* (70 satDNAs) and *P. semifasciata* (71 satDNAs) (Table S4). Such a scenario (i.e., the conservation and sharing of satDNA families) is not an exclusive feature of *Pyrrhulina* but is also observed in other fish species [39,40,41], as well as several vertebrates such as snakes [42], primates [43], and true toads [44]. According to the library hypothesis [45], satellite sequences are preserved over long evolutionary timescales because closely related species share a common library of these sequences, with quantitative changes brought on by differential amplification. Investigations suggested that the satDNA libraries can disappear or be formed de novo following cladogenetic events [46]. According to this theory, the acquisition of a biological function that will ultimately be preserved by natural selection is necessary for conservation over a lengthy evolutionary time. Although both species analyzed retained about half of their satDNA families, evidencing a common library between them, the observed differences in the abundance of these shared families are also remarkable, a fact predicted by the library hypothesis [45]. Among the 38 shared satDNAs, six of them were selected for FISH experiments (Table S4) and revealed the same accumulation pattern in both species.

The heterochromatic regions differ substantially between *Pyrrhulina* species, with large accumulations in centromeric and telomeric regions, as well as pericentromeric and interstitial regions [28,29,30]. The karyotype of *P. semifasciata* has considerable accumulations of heterochromatin in centromeres and telomeres [29], whereas *P. marilynae* has these accumulations mainly in the centromeric region of most chromosomes [30]. In the same way, the in situ mapping revealed a predominance of PseSatDNAs in the centromeric regions of *P. semifasciata* (Figure 2). This type of association can be suggestive of a probable relationship with centromere development, as well as an essential role in genome integrity by preserving the higher-level nucleus structure [47]. Although satellite DNA sequences are often abundant in heterochromatic regions [10,48], as indicated in *P. semifasciata*, their occurrence in euchromatic regions has been emphasized in many species, including bivalves [49], insects [13,17,50], mammals [51], and fishes [39,40,52], as well as *P. marilynae* (Figure 1). PmaSat01 constitutes approximately 4% of the *P. marilynae* genome (Table S1), while the most abundant satellite of *P. semifasciata* (PseSat01) only represents about 0.5% of its genome (Table S2). However, the satellitomes of both species are very similar in the number of clusters recovered by TAREAN (i.e., 70 PmaSatDNAs and 71 PseSatDNAs). Among fishes, other Characiformes species, such as representatives of *Astyanax*, *Characidium*, and *Psalidodon*, also carry similar numbers of satDNA families [41,53]. However, species with more satDNAs have previously been identified, such as in *Megaleporinus*, demonstrating a great dynamic of such sequences in fish genomes. Owing to the various processes involved in their dynamics throughout cladogenetic genomic development, the variety of satellitomes among different species of animals and plants is rather significant, both in terms of the number of satDNA families and the proportion of the genome they occupy [10,54].

### 3.2. Satellite DNA Contribution in the Significant 2n Reduction Observed in P. marilynae

*P. marilynae* stands out for presenting the most rearranged karyotype among all *Pyrrhulina* species, with 2*n* = 32 and formed by large and unusual metacentric pairs [30]. Many studies have found that satellite DNAs can induce chromosomal rearrangements and thus have a direct impact on karyotype evolution due to their dynamic and fast-evolving nature [55,56,57,58,59]. Thus, the occurrence of centric fusions and fissions is frequently associated with the fast dynamics of satDNAs, which are located mostly in the centromeric and pericentromeric regions of chromosomes [60,61,62,63]. Interestingly, our experiments demonstrate that PmaSatDNAs hybridized at telomeres or centromeres of *P. marilynae* chromosomes, including bitelomeric signals from PmaSat04 and PmaSat10, and pericentromeric signals on both arms with PseSat01 (Figure 1). These results imply that chromosomal reduction in *P. marilynae* is directly linked to centric fusion processes that result in the large metacentric chromosomes found in this species’ karyotype. Notably, the satellite DNA sequences did not experience any accumulation or loss after the fusion process.

PmaSatDNAs 04, 07, 09, and 10 accumulate in telomeres and (peri)centromeres, especially in the large metacentric pairs. These large chromosomes probably originate from chromosome fusions, restricting recombination to the distal ends [64,65,66]. Repetitive sequences, such as satellites and rDNAs, often make up the subtelomere region, also known as the buffer zone between the internal chromosome and telomere. Given that the latter is made up of telomeric motifs interspersed with other repeated sequences [67], such subtelomere sequences can also be found in interstitial telomeric sites (ITSs). As sub and telomeric arrays help to stabilize new ends [68], it is likely that the FISH signals observed directly represent the fusion process that *P. marilynae* went through throughout its karyotypic reduction (Figure 1). The expansion and contraction of those telomere-associated satellite motifs that remained even after the inactivation of the telomeric region near the newly formed centromere may be fostered by repair mechanisms [69]. This scenario is not exclusive to *P. marilynae*. Other fish species also carry a series of repetitive DNA in their chromosome fusion points, as observed in *Rineloricaria* [70,71], for example.

SatDNAs are a significant and prominent component of the so-called “dark matter of genomes” [72]. In fact, multiple pieces of evidence show that satellite DNAs are sequences that can participate in centromere and telomere formation besides presenting fundamental roles and specific functions in the genome [40,54,73,74]. Although no functional experiments have been performed here, the majority of the PmaSatDNAs are found in centromeric and telomeric regions and may be directly linked to their formation, in addition to possibly playing a role in *P. marilynae*’s reduction and karyotypic evolution.

### 3.3. SatDNAs and the Evolution of Multiple Sex Chromosomes

Repetitive sequences are great tools for the study of sex chromosomes, where species with heteromorphic sex chromosomes show a difference in the accumulation of some sequences between males and females, emphasizing the existence of several W/Y-specific satDNAs [13,14,18,75]. Despite the presence of a well-differentiated X_1_X_2_Y sex chromosome system, minor differences in haplotype accumulation between males and females were observed (Figure 6). Although four PseSatDNAs were mapped in this study to either X_1_, X_2_, or neo-Y chromosomes, no neo-Y-specific satDNA was identified (Figure 5), contrasting with *Eneoptera surinamensis* [76] and *Ronderosia bergii* [14], which show a large accumulation of satDNAs in neo-sex chromosomes. These species, however, lack synapses between sex chromosomes, demonstrating significant differentiation between them. Multiple-sex chromosome systems, in contrast to simple ones, are known to have a more recent origin and still exhibit considerable recombination rates, resulting in few or no sex-specific sequences [75]. A similar scenario has already been reported in other animal species, such as the frog *Proceratophrys boiei*. Despite having a simple and heteromorphic ZZ/ZW sex chromosome system, it is distinguished by the absence of sex-specific satDNAs and low sex divergence, indicating their early stage of sex chromosome differentiation [77].

There are no dated phylogenetic reconstructions for *Pyrrhulina* species. As a result, determining when the multiple-sex chromosome system emerged is not feasible, regardless of other cytogenetic traits that suggest its recent origin. The X_1_, X_2_, and Y sex chromosomes lack strong differences in size and accumulation of heterochromatic regions [29], as in the satellite distribution. Accordingly, the MST generated for PseSat55 also did not point to any difference in the abundance of the haplotypes obtained from reads of females and males (Figure 6). Such low heterochromatin is also observed in other species with recent diversification of multiple sex chromosomes, such as *Hoplias malabaricus* and *Erythrinus erythrinus* [78,79]. Considering the formation of a trivalent during the meiosis process, multiple-sex chromosome systems (such as the X_1_X_2_Y of *P. semifasciata*) could not accumulate large heterochromatic blocks; otherwise, they could impair its correct segregation [80]. In this sense, the presence of PseSatDNAs in the homologous regions of the multiple sex chromosomes of *P. semifasciata* can be indicative of their role in the modulation of gene expression. Under stress circumstances, euchromatic copies of pericentromeric satDNAs in *Tribolium castaneum* are functionally relevant in modifying chromatin and the expression of adjacent genes [81]. In *Drosophila melanogaster*, more than a thousand euchromatic copies of satDNAs are mainly found near genes and are thought to have a function in the modulation of gene expression [82]. Similarly, those euchromatin-dominant satellite DNAs share characteristics regarding their structure, organization, and evolution (reviewed in [10,83]).

Despite the absence of neo-Y-specific satDNA sequences in *P. semifasciata*, we demonstrate that several motifs are shared between this species’ sex chromosome and the proto-sex pairs in other *Pyrrhulina* representatives (Figure 5). Considering the absence of specific sequences such as PseSat38 in *P. obermulleri*, it is also noteworthy to emphasize the high dynamism of these sequences in the cladogenetic history. Both a seemingly complete absence of these sequences and their low-copy numbers can be explained by the absence of detectable FISH signals. Unequal crossing over between sister chromatids or intra and interchromosomal recombination may significantly decrease the copy number of satellite DNAs [84]. On the other hand, sequences such as PseSat01, PseSat04, and PseSat55 suffered expansions and retractions in the number of loci, including changes between both proto-sex pairs. In some instances, site-directed recombination between homologous motifs in satellite repeats and in the target genomic sequence, most likely mediated by extrachromosomal circular DNA, can explain the dispersion of satellites [85]. Alternatively, such translocation can be due to transposable elements (TEs) that are known to participate in the origin and the dispersal of satDNA repeats [86]. Through amplifying tandem repeats within them, TEs may also capture satDNA motifs and generate new satDNAs, which can then be disseminated over other regions of the genome and preserved through nonreciprocal transfer mechanisms such as unequal crossover [87]. Indeed, TEs such as Rex3 were already mapped in *Pyrrhulina* chromosomes, showing a high dispersal tendency [28]. However, the extremely similar size and shape of those chromosomes, as well as the frequent translocations between them, might also be the result of putative pseudo-homologous regions (PHRs), which are thought to be due to recombination between non-homologous sequences [88] or due to the presence of pseudoautosomal regions (PARs) with high recombination rates [89] in these proto-sex chromosomes.

It is noteworthy that some conserved PseSatDNAs across distinct *Pyrrhulina* species (Figure 4) are present in the pericentromeric region of the sex chromosomes in *P. semifasciata* and the proto-sex pairs of *P. brevis*, *P. obermulleri*, and *P. marilynae*. From the canonical model of sex chromosome evolution, when the recombination between the sex-related chromosomes is ceased, the sex-specific chromosome is invaded by several repetitive sequences, including satDNAs (reviewed in [90]). However, the presence of such satDNAs in the proto-sex chromosomes leads us to two main questions: i) Can the accumulation of the same PseSatDNAs (i.e., PseSat01, PseSat04, PseSat38, and PseSat55) in proto-sex and multiple-sex chromosomes indicate the presence of a plesiomorphic and homomorphic XX/XY in *P. brevis*, *P. obermulleri*, and *P. marilynae*? Or ii) Were those sequences simply conserved in these acrocentric pairs, given their putative role in the centromeric structure and genome integrity? To fully solve these questions, however, a high-quality reference genome is mandatory, and the next steps involve generating a high-quality genome assembly with PacBio having access to an end-to-end solution.

## 4. Materials and Methods

### 4.1. Material, Mitotic Chromosomes and DNA Sequencing

Samples of four *Pyrrhulina* species were collected in Brazilian rivers, according to Table 1. To obtain mitotic metaphase chromosomes, the animals were euthanized with eugenol, as approved by the Ethics Committee on Animal Experimentation of the Universidade Federal de São Carlos, Brazil (Process number CEUA 7994170423), and cell suspensions were obtained from kidney cells [91]. For DNA sequencing, we selected *P. marilynae* (one male) and *P. semifasciata* (one male and one female) individuals. Then, total DNA was extracted from muscle tissues using a spin-column-based protocol (Cellco Biotech, São Carlos–SP, Brazil). The purified DNAs were then sequenced in the BGISEQ-500 platform (2 × 150 bp; BGI Shenzhen Corporation, Shenzhen, China). Short-read sequencing yielded between 2.14 Gb (in *P. semifasciata* female) and 2.44 Gb (in *P. marilynae*). Raw reads were deposited in the sequence read archive (SRA-NVBI) and are available under the accession numbers (SRR25467276, SRR25476502, and SRR25476501).

### 4.2. Bioinformatic Analyses and satDNA Library

Firstly, we trimmed the raw reads with Trimmomatic [92] to select the pair-end reads with Q > 20 for all nucleotides. Then, the satDNA catalogs from *P. semifasciata* and *P. marilynae* were independently characterized on TAREAN [93] with the satMiner pipeline [12]. Specifically, the consensus satDNA sequences outputted in TAREAN were filtered from the genomic libraries with DeconSeq [94], and subsequent iterations were performed on TAREAN until no satDNA was found. Then, a homology search with RepeatMasker [95] was performed to group the sequences into variants, families, and superfamilies, as suggested by [12]. We also calculated the abundance and divergence values of the satDNA families by selecting 10,000,000 reads (2 × 5,000,000) from each genomic library and masking against their own catalog of satDNAs with RepeatMasker [95]. After that, we named the satDNA families according to their abundance in each species.

Since *P. semifasciata* exhibits a multiple-sex chromosome system, we calculated the male: female (M/F) abundance ratio of each satDNA family in this species (quotient between the abundance of the same satellite DNA in male and female). Then, we selected those with an M/F ratio greater than 1.2 as putatively sex-specific accumulating satDNAs. Finally, both satellitomes were BLAST-searched [96] against the NCBI nucleotide collection to check for the presence of conserved satDNAs. We constructed a minimum spanning tree (MST) for PseSat55 using PHYLOVIZ [97] to describe the proportions of the male and female haplotypes.

### 4.3. Primer Design and DNA Amplification via Polymerase Chain Reaction (PCR)

We designed primers for 21 out of the 70 PseSatDNAs and for 10 of the 71 PmaSatDNAs that were characterized. As a criterion for primer selection, we selected the ten most abundant ones (in *P. marylinae*, Table S1), the five most abundant, and those with some difference in abundance between sexes (in *P. semifasciata*, Table S2). PCR procedures used the optimal amplification temperatures and DNA template concentrations for each satDNA, according to [12]. For each sequence, the following cycles were used: initial denaturation at 95 °C for 5 min; 29–35 cycles with denaturation at 95 °C for 15 s; annealing at 50 °C to 62 °C for 30 s (Table S3); extension at 72 °C for 10 s, and final extension at 72 °C for 10 min. To validate the amplification and ensure the integrity of the satDNAs, the PCR products were checked by electrophoresis on 2% and 1% agarose gels. Finally, they were quantified using the NanoDrop spectrophotometer (ThermoFisher Scientific, Branchburg, NJ, USA).

### 4.4. Fluorescence In Situ Hybridization (FISH)

The probes derived from the satDNA’s PCRs were labeled with Atto550-dUTP or Atto488-dUTP by Nick-Translation (Jena Biosciences, Jena, Germany) and used for FISH experiments. The hybridization mixes were composed of 100 ng of each labeled satellite DNA plus 50% formamide, 2 × SSC, 10% SDS, 10% dextran sulfate, and Denhardt’s buffer at pH 7.0 in a total volume of 20 µL, following high-stringency conditions for FISH [98]. We hybridized the above-mentioned selected sequences in both species (i.e., *P. marilynae* and *P. semifasciata*), then selected those satDNAs that displayed positive signals on the sex chromosomes of *P. semifasciata* to hybridize in *P. brevis* and *P. obermulleri* chromosomes. For the satDNA FISH experiments, glass slides containing metaphase chromosomes were aged for 1 h at 60 °C, following a treatment at 37 °C for 5 min with 0.005% pepsin solution (99 µL H_2_O, 10 µL HCl, and 2.5 µL pepsin (20 mg/mL). Chromosomes were denatured in 70% formamide/2 × SSC at 72 °C for 3 min, while probes were at 85 °C for 10 min, then cooled at 4 °C for 2 min before application. Hybridization occurred overnight in a moist chamber at 37 °C. Next, the slides were washed for 5 min in 1 × SSC at 65 °C and 4 × SSC/Tween at room temperature, following a quick wash in 1 × PBS for 1 min. The slides were dehydrated in an ethanol row (70%, 85%, and 100%) before the counterstaining of the chromosomes with DAPI mounted in Vectashield (Vector Laboratories, Burlingame, USA). We also used whole-chromosome painting (WCP) using a Y-specific probe (Psemi-Y) previously obtained [29] to detect the sex chromosomes of *P. semifasciata* and the proto-sex pairs in *P. brevis* and *P. obermulleri*. For this, sequential FISH/WCP was performed following [99]. In total, 10 PseSatDNAs showed visible FISH signals in *P. semifasciata,* and 07 PmaSatDNA showed a visible FISH signal in *P. marilynae*.

### 4.5. Images and Confirmation of Results

To confirm the FISH results, we analyzed a minimum of 30 metaphase spreads per individual. Images were captured with CoolSNAP on an Axioplan II microscope (Carl Zeiss Jena GmbH, Jena, Germany) and processed with ISIS (MetaSystems Hard & Software GmbH, Altlussheim, Germany).

## Figures and Tables

**Table 1:** Species, locality, and number of individuals (*N*) used in the present study.

Species	Locality	*N*	Voucher
*P. brevis*	Adolfo Ducke Reserve- Igarapé Barro Branco, Manaus–AM (2°56′04.6″ S 59°58′10.6″ W)	04♂; 07♀	MZUSP 123077
*P. marilynae*	Ipiranga do Norte–MT (11°36′02″ S 55°56′27″ W)	13♂; 04♀	UFPB 12080
*P. obermulleri*	Tefé-AM (3°25′50.7″ S 64°44′54.8″ W)	06♂; 04♀	UFPB 12079
*P. semifasciata*	Adolfo Ducke Reserve- Igarapé Barro Branco, Manaus–AM (2°56′04.6″ S 59°58′10.6″ W)	07♀; 12♂	MZUSP 123080

MT = Mato Grosso and AM = Amazonas Brazilian States.

## Data Availability

The datasets generated during and/or analyzed during the current study are available from the corresponding author upon reasonable request. The datasets generated and analyzed during the current study are available in the GenBank repository under the accession numbers OR094701-OR094771 and OR094772-OR094841.

## Supplementary Materials

The following supporting information can be downloaded at https://www.mdpi.com/1422-0067/24/17/13654/s1.
