# Supplementary material for: Chromosomal Rearrangements and Satellite DNAs: Extensive Chromosome Reshuffling and the Evolution of Neo-Sex Chromosomes in the Genus Pyrrhulina (Teleostei; Characiformes)
Source: Int J Mol Sci. 2023 Sep 4;24(17):13654. doi: 10.3390/ijms241713654 (PMC10563077; doi:10.3390/ijms241713654)
Supplement: Supplementary file 1 [file ijms-24-13654-s001.zip › Table S1.pdf]

Supplementary Table S1. Main characteristics of 71 satDNAs found in *Pyrrhulina marilynae*. The sequences highlighted in blue correspond to those selected for the FISH experiments.

Pyrrhulina marilynae				
SatDNA family	RUL	Abundance	Divergence	A+T (%)
PmaSat01-1627	1627	0.0395879278883	1.33	58.6
PmaSat02-68	68	0.0126290012853	2.63	66.2
PmaSat03-45	45	0.0083896090511	2.92	44.4
PmaSat04-50	50	0.00772308401902	3.93	60
PmaSat05-226	226	0.00728803566553	9.69	64.2
PmaSat06-198	198	0.00647761332963	9.25	67.2
PmaSat07-45	45	0.00639111613516	5.97	62.2
PmaSat08-33	33	0.00268802096213	2.10	57.6
PmaSat09-335	335	0.00220671410467	9.24	70.1
PmaSat10-4663	4663	0.00209498220866	5.93	43.6
PmaSat11-2486	2486	0.00207120592574	10.40	62.4
PmaSat12-842	842	0.00204339498168	9.60	63.5
PmaSat13-4322	4322	0.00196875721573	13.26	63.7
PmaSat14-54	54	0.00185443026527	3.79	57.4
PmaSat15-1437	1437	0.0015994228488	8.96	58.1
PmaSat16-670	670	0.000982230583972	7.52	59.4
PmaSat17-192	192	0.000746618253954	10.82	63
PmaSat18-42	42	0.000666838894057	4.21	64.3
PmaSat19-84	84	0.000628643771793	13.99	53.6
PmaSat20-142	142	0.000590982313911	3.14	66.2
PmaSat21-712	712	0.000582114970286	1.67	62.2
PmaSat22-32	32	0.000555307988527	3.71	53.1
PmaSat23-23	23	0.000553234717251	7.65	52.2
PmaSat24-1283	1283	0.000550808970056	13.84	56.5
PmaSat25-36	36	0.000541725784436	5.34	44.4
PmaSat26-21	21	0.000528704967553	17.33	47.6
PmaSat27-39	39	0.000519611880924	8.11	64.1
PmaSat28-165	165	0.000493380147786	7.48	66.7
PmaSat29-51	51	0.000480608836328	7.36	39.2
PmaSat30-1062	1062	0.00047705239391	8.96	64.8
PmaSat31-47	47	0.000450014718642	5.81	59.6
PmaSat32-33	33	0.000425481998641	3.84	60.6
PmaSat33-176	176	0.00040900473953	4.45	64.8
PmaSat34-1411	1411	0.000394930455293	0.99	58
PmaSat35-845	845	0.000394410652323	3.59	62.7
PmaSat36-445	445	0.00032788478302	4.71	57.1
PmaSat37-28	28	0.000311260988976	9.21	60.7
PmaSat38-162	162	0.000284214402801	4.71	66
PmaSat39-88	88	0.000276674784478	8.15	68.2
PmaSat40-211	211	0.000276362902695	6.43	59.2
PmaSat41-915	915	0.000237961839444	1.67	67.4
PmaSat42-314	314	0.000236262826307	5.27	58.3
PmaSat43-165	165	0.00023088459824	4.09	64.8
PmaSat44-43	43	0.000227744988298	11.66	48.8

PmaSat45-568	568	0.000214584567189	5.78	59
PmaSat46-470	470	0.000206393462476	5.08	61.3
PmaSat47-672	672	0.000203039000641	3.93	58.5
PmaSat48-31	31	0.000193220170055	5.51	67.7
PmaSat49-80	80	0.00017571122581	17.60	67.5
PmaSat50-428	428	0.000172995379053	7.23	57.2
PmaSat51-414	414	0.000172949834411	7.17	61.6
PmaSat52-387	387	0.000167174575885	4.40	58.1
PmaSat53-36	36	0.000162056744354	7.38	61.1
PmaSat54-44	44	0.000156811189807	4.92	61.4
PmaSat55-231	231	0.000144396314672	6.74	55
PmaSat56-544	544	0.000139795315808	4.75	58.8
PmaSat57-98	98	0.00013769828211	7.53	61.2
PmaSat58-165	165	0.000130510149605	6.24	63.6
PmaSat59-167	167	0.000125421031	6.72	70.7
PmaSat60-31	31	0.000124081424488	7.00	51.6
PmaSat61-142	142	0.000117378441422	8.02	71.8
PmaSat62-47	47	0.000115912101995	4.91	59.6
PmaSat63-33	33	0.000114132890685	2.66	60.6
PmaSat64-39	39	0.0001140388311	5.89	66.7
PmaSat65-64	64	0.000106678421039	2.74	54.7
PmaSat66-188	188	0.000101115044104	8.31	66.5
PmaSat67-39	39	0.00000989358	4.82	53.8
PmaSat68-173	173	0.00000899655	8.21	62.4
PmaSat69-53	53	0.0000078606	4.77	56.6
PmaSat70-33	33	0.00000701149	4.78	51.5
