# Supplementary material for: Chromosomal Rearrangements and Satellite DNAs: Extensive Chromosome Reshuffling and the Evolution of Neo-Sex Chromosomes in the Genus Pyrrhulina (Teleostei; Characiformes)
Source: Int J Mol Sci. 2023 Sep 4;24(17):13654. doi: 10.3390/ijms241713654 (PMC10563077; doi:10.3390/ijms241713654)
Supplement: Supplementary file 1 [file ijms-24-13654-s001.zip › Table S2.pdf]

Supplementary Table S2. Main characteristics of 70 satDNAs found in *Pyrrhulina semifasciata*, highlighting, in blue, those chosen for FISH mapping. The asterisk (*) indicates the satDNAs mapped in the sex chromosomes.

Pyrrhulina semifasciata

SatDNA family	RUL	Abundance (M)	Abundance (F)	Abundance (M/F)	Divergence (M)	Divergence (F)	A+T (%)
PseSat01- 304*	304	0,005606905	0,006386826	0,877885927	9,78	9,65	68
PseSat02- 45	45	0,005289071	0,00496321	1,065655359	4,97	4,98	62,2
PseSat03- 68	68	0,005288433	0,005331731	0,991879186	2,52	2,54	66,1
PseSat04-226*	226	0,004975278	0,005546355	0,897035567	9,76	9,62	64,6
PseSat05 - 45	45	0,004215905	0,004087883	1,03131743	4,4	4,43	44,4
PseSat06- 198	198	0,002799141	0,003195114	0,876069106	8,88	8,81	66,6
PseSat07- 174	174	0,002764281	0,002730314	1,012440816	4,55	4,66	62,6
PseSat08- 2005	2005	0,002341543	0,00191567	1,222310384	10,37	10,38	62,3
PseSat09- 50	50	0,002080652	0,001881225	1,10600892	8,37	9,01	60
PseSat10- 610	610	0,001957573	0,001948731	1,004537311	16,83	16,92	66
PseSat11-2510	2510	0,001864105	0,001601655	1,163861305	12,58	11,71	62
PseSat12- 2219	2219	0,001725149	0,001426195	1,209616448	3,71	3,84	63,8
PseSat13- 35	35	0,001527915	0,001394119	1,095972222	1,86	1,8	57,1
PseSat14- 1235	1235	0,001207345	0,001038919	1,162116533	11,49	11,91	57,4
PseSat15- 38	38	0,001082321	0,001210707	0,893957307	13,9	14,32	63,1
PseSat16- 42	42	0,00096017	0,000568407	1,689228734	7,36	7,61	59,5
PseSat17- 381	381	0,000850538	0,000685979	1,23988987	8,74	8,25	61,4
PseSat18- 615	615	0,000729584	0,000612211	1,191720497	6,62	6,4	58,2
PseSat19- 461	461	0,000715265	0,000645695	1,10774328	11,51	9,8	63,5
PseSat20- 1284	1284	0,000705413	0,00074095	0,952039049	20,91	20,17	56,3
PseSat21- 42	42	0,000696777	0,000711353	0,979510398	4,22	4,15	64,2
PseSat22- 54	54	0,000664187	0,000593948	1,118258389	6,05	6,44	57,4
PseSat23- 155	155	0,000658736	0,000516891	1,274418737	6,28	6,5	63,8
PseSat24- 84	84	0,000643957	0,000633686	1,016208869	14,6	14,94	0,535714
PseSat25- 52	52	0,000643279	0,000613842	1,047954794	13,95	14,18	0,557692
PseSat26- 828	828	0,000613564	0,000594593	1,03190528	7,57	7,25	0,509662
PseSat27- 422	422	0,000581057	0,000722512	0,804218246	6,35	6,6	0,575829
PseSat28- 14	14	0,000557749	0,000571732	0,975542154	6,92	6,86	0,785714
PseSat29- 6	6	0,000552197	0,000573221	0,963321905	26,22	26,24	0,5
PseSat30- 192	192	0,000529303	0,0005363	0,986953819	15,32	15,9	0,640625
PseSat31- 51	51	0,000441051	0,000393715	1,120227305	9,09	9,1	0,392157
PseSat32- 186	186	0,000390964	0,000658971	0,593294997	8,44	8,02	0,709677
PseSat33- 880	880	0,000336683	0,000305781	1,101059365	3	3,46	0,669318
PseSat34-165	165	0,000282004	0,000328703	0,857928629	6,61	6,58	0,642424
PseSat35- 23	23	0,000282019	0,000250551	1,125598016	11,53	12,52	0,521739
PseSat36- 195	195	0,000237211	0,000183166	1,295058399	3,36	3,42	0,707692
PseSat37- 1041	1041	0,000231577	0,000229568	1,008752672	10,1	9,49	0,623439
PseSat38- 300*	300	0,000230661	0,000059944	3,847935851	4,96	6,47	0,576667
PseSat39- 80	80	0,000225943	0,000317697	0,711188427	15,55	15,44	0,6625
PseSat40- 39	39	0,000215363	0,000275735	0,781050911	10,74	9,56	0,641026
PseSat41- 21	21	0,000203179	0,000205974	0,986431944	17,77	16,97	0,47619

PseSat42- 1093	1093	0,000198728	0,000179851	1,104957057	1,5	1,56	0,600183
PseSat43- 124	124	0,000194009	0,000155268	1,249512671	7,66	8,11	0,564516
PseSat44- 28	28	0,00019343	0,000196448	0,984637156	9,47	9,48	0,571429
PseSat45- 445	445	0,00018569	0,000175259	1,059515613	3,95	4,07	0,573034
PseSat46- 837	837	0,000176346	0,000178717	0,986731375	7,57	7,21	0,624851
PseSat47- 21	21	0,000174861	0,000236993	0,737829475	11,25	11,75	0,666667
PseSat48- 32	32	0,000171967	0,000124967	1,376098287	5,94	7,75	0,53125
PseSat49- 33	33	0,000170543	0,000131041	1,301441784	6,45	7,5	0,606061
PseSat50- 1125	1125	0,000160486	0,000123408	1,300450538	9,97	12,7	0,656
PseSat51- 713	713	0,000146761	0,00014616	1,004109652	5,34	5,72	0,622721
PseSat52- 673	673	0,000141336	0,000132213	1,069004987	7,81	7,72	0,576523
PseSat53- 23	23	0,000138389	0,000075714	1,827790545	8,88	10,24	0,565217
PseSat54- 159	159	0,00013754	0,000194867	0,705815943	5,1	4,7	0,672956
PseSat55- 43*	43	0,000133589	0,000159353	0,838325057	8,62	7,99	0,488372
PseSat56- 87	87	0,000126526	0,000159655	0,792494666	6,25	5,81	0,678161
PseSat57- 162	162	0,000124983	8,86007E-05	1,410636489	4,99	5,06	0,67284
PseSat58- 372	372	0,000122859	0,00012864	0,955063226	2,88	2,95	0,567204
PseSat59- 46	46	0,000116657	0,000119628	0,975167464	12,34	12,69	0,608696
PseSat60- 944	944	0,000116336	0,000101468	1,146528955	7,8	7,73	0,610169
PseSat61- 213	213	0,000109063	0,000137659	0,792264963	5,14	5,29	0,615023
PseSat62- 469	469	0,000103981	0,000108045	0,962379989	4,37	3,96	0,614072
PseSat63- 463	463	0,000100169	0,000113188	0,884981918	3,94	3,69	0,591793
PseSat64- 182	182	0,000098656	5,97373E-05	1,651496552	10,95	13,61	0,681319
PseSat65- 187	187	9,83113E-05	8,62127E-05	1,14033514	5,38	5,61	0,620321
PseSat66- 44	44	8,66047E-05	0,000077618	1,115780704	4,41	4,93	0,613636
PseSat67- 198	198	8,07013E-05	0,000103397	0,780502273	4,81	4,26	0,60101
PseSat68- 179	179	7,91173E-05	7,52593E-05	1,051262745	6,5	6,33	0,614525
PseSat69- 592	592	7,38767E-05	7,23727E-05	1,020781326	4,78	4,41	0,586149
PseSat70- 759	759	7,23553E-05	6,96727E-05	1,038503861	4,52	4,48	0,586298
PseSat71- 182	182	6,96327E-05	6,92727E-05	1,005196855	6,83	7,08	0,538462
