# Supplementary material for: Chromosomal Rearrangements and Satellite DNAs: Extensive Chromosome Reshuffling and the Evolution of Neo-Sex Chromosomes in the Genus Pyrrhulina (Teleostei; Characiformes)
Source: Int J Mol Sci. 2023 Sep 4;24(17):13654. doi: 10.3390/ijms241713654 (PMC10563077; doi:10.3390/ijms241713654)
Supplement: Supplementary file 1 [file ijms-24-13654-s001.zip › Table S3.pdf]

Supplementary Table S3. PCR conditions (primer, temperature, and concentration of template DNA) for the optimal amplification of satellite DNAs from *P. marilynae* and *P. semifasciata*.

Satellite	Primer F	Primer R	Anneraling temperature range	[DNA] ng/µl
PmaSat01-1627	5'CACACCTTTGGCATTC TAGC	5'TCTGTTAAGCAT GGTGGAGG	56°C – 57,4°C	100
PmaSat04-50	5'GGGTGTGGTTATCTCT GTAC	5'CCCCTCTAAAC AGAGTATAAAC	50°C – 51,7°C	100; 10; 1; 0,1
PmaSat05-226	5'GCAAGCTGAATACAT TCATG	5'GACTGTCTGAG AGCACAAAC	53°C - 54,5°C	100; 10; 1; 0,1
PmaSat06-198	5'AAGACAGCTTCTGCA TCCATG	5'TTGCAGATTTGC CCAAAAAC	55°C - 55,6°C	100; 10; 1; 0,1
PmaSat07-45	5'CCTCTGTAACACATTA AACTGT	5'TAGGAGAGTGT AGTGTTAGTCT	51,7°C – 58°C	100; 10
PmaSat09-335	5'CTGCAACCACTTCCA GTGAT	5'ACACTTAGTTGT GTCTGAAA	53°C - 53,5°C	100; 10; 1; 0,1
PmaSat10-4663	5'AGAACGGAGGTCTCT TGCGT	5'CTCCATTCATTT CCATCACGC	56°C – 62,5°C	100
PseSat01-304	5'CTGCAACCACT TCCAGTGAT	5'ACACTTAGTTGT GTCTGAAA	53°C - 53,5°C	100; 10; 1; 0,1
PseSat04-226	5'GCAAGCTGAAT ACATTCATG	5'GACTGTCTGAG AGCACAAAC	53°C - 54,5°C	100; 10; 1; 0,1
PseSat06-198	5'AAGACAGCTTC TGCATCCATG	5'TTGCAGATTTGC CCAAAAAC	55°C - 55,6°C	100; 10; 1; 0,1
PseSat32-186	5'TGAGAGAAGAC TTTACAAGC	5'TGACACATTTA AGGCATTTC	50°C - 54,9°C	100
PseSat34-165	5'CATGGCTGATAG GGTAAAAG	5'ATGTGACCACA CTGTATCCC	56,5°C - 63°C	1; 0,1
PseSat38-300	5'ATAGACGGATGG ATTAACGG	5'AAAGACTGACA GACCATTAT	53°C - 61°C	100; 10; 1; 0,1
PseSat39-80	5'CTTACTGTCTCAT ATCTGTG	5'AGTTAAGACCA GTATCTCTA	50,5°C - 57,4°C	100
PseSat50-1125	5'CTGTACAGTGGA TTATGGAG	5'TTCGTGTTACTC AGAATGTC	53 - 53,5	100; 10
PseSat54-159	5'AATCTCTGCATAT AAAATGGC	5'AGATGGACCAA AAGGTGTAT	50°C - 58°C	100; 10; 1; 0,1
PseSat55-43	5'CTGTGGTGCACTA ACCAGA	5'CCCTATCATTTA CAGTACA	50°C - 58°C	100; 10; 1; 0,1
PseSat56-87	5'CACCCAGCCACTT TTTTA	5'TGGAGGTAGTT AGTTAGATG	50°C - 51,5°C	100
PseSat57-162	5'AGGGTCAGTACTC TCACT	5'CACTTCATAAC AGTCATTTTAA	52°C - 60°C	100; 10; 1; 0,1
PseSat61-213	5'ATTCCAGGCAATAA TCTGCC	5'CAGGCGAGAAT TCTACCACT	53°C - 57,9°C	100; 10; 0,1
PseSat63-463	5'ATTGGTCAGATATT GTGAAG	5'ATTGCGCCGTTC ATATTCAC	52°C - 60°C	100; 10
PseSat64-182	5'TTTACTGCTGTTAAGA TTTC	5'CCTTTGGTAGG ACATGTAGC	50°C - 50,7°C	100; 10; 1; 0,1
PseSat67-198	5'CTAACTTCATTCGGCG TTCT	5'CCACCATGGCA CACCTGATA	55°C - 55,6°C	10; 1; 0,1
