# Supplementary material for: Chromosomal Rearrangements and Satellite DNAs: Extensive Chromosome Reshuffling and the Evolution of Neo-Sex Chromosomes in the Genus Pyrrhulina (Teleostei; Characiformes)
Source: Int J Mol Sci. 2023 Sep 4;24(17):13654. doi: 10.3390/ijms241713654 (PMC10563077; doi:10.3390/ijms241713654)
Supplement: Supplementary file 1 [file ijms-24-13654-s001.zip › Table S4.pdf]

Supplementary Table S4. Shared SatDNA between *P. marilynae* and *P. semifasciata*. The sequences highlighted in blue correspond to those selected for the FISH experiments

Pyrrhulina marilynae	*Pyrrhulina semifasciata*	Classification
PmaSat02-68	PseSat03-68	SV
PmaSat03-45	PseSat05-45	SV
PmaSat04-50	PseSat09-50	SV
PmaSat05-226	PseSat04-226	SV
PmaSat07-45	PseSat02-45	SV
PmaSat08-33	PseSat13-35	V
PmaSat11-2486	PseSat11-2510	V
PmaSat14-54	PseSat22-54	SV
PmaSat17-192	PseSat30-192	SV
PmaSat18-42	PseSat21-42	SV
PmaSat19-84	PseSat24-84	SV
PmaSat21-712	PseSat51-713	SV
PmaSat22-32	PseSat48-32	SV
PmaSat23-23	PseSat35-23	SV
PmaSat24-1283	PseSat28-1284	V
PmaSat26-21	PseSat47-21	SV
PmaSat27-39	PseSat40-39	SV
PmaSat28-165	PseSat54-159	V
PmaSat29-51	PseSat31-51	SV
PmaSat32-33	PseSat49-33	SV
PmaSat35-845	PseSat46-837	V
PmaSat36-445	PseSat45-445	V
PmaSat37-28	PseSat44-28	SV
PmaSat38-162	PseSat57-162	SV
PmaSat39-88	PseSat56-87	SV
PmaSat40-211	PseSat61-213	V
PmaSat41-915	PseSat33-880	V
PmaSat44-43	PseSat55-43	SV
PmaSat45-568	PseSat69-592	V
PmaSat46-470	PseSat62-469	V
PmaSat47-672	PseSat52-673	V
PmaSat49-80	PseSat39-80	SV
PmaSat52-387	PseSat58-372	V
PmaSat54-44	PseSat66-44	SV
PmaSat12-842	PseSat15-38	SF
PmaSat15-1437	PseSat14-1235	SF
PmaSat66-188	PseSat06-198	SF
PmaSat55-231	PseSat71-182	SF
