# Supplementary figures and images for: Chromosomal Rearrangements and Satellite DNAs: Extensive Chromosome Reshuffling and the Evolution of Neo-Sex Chromosomes in the Genus Pyrrhulina (Teleostei; Characiformes)
Source: Int J Mol Sci. 2023 Sep 4;24(17):13654. doi: 10.3390/ijms241713654 (PMC10563077; doi:10.3390/ijms241713654)

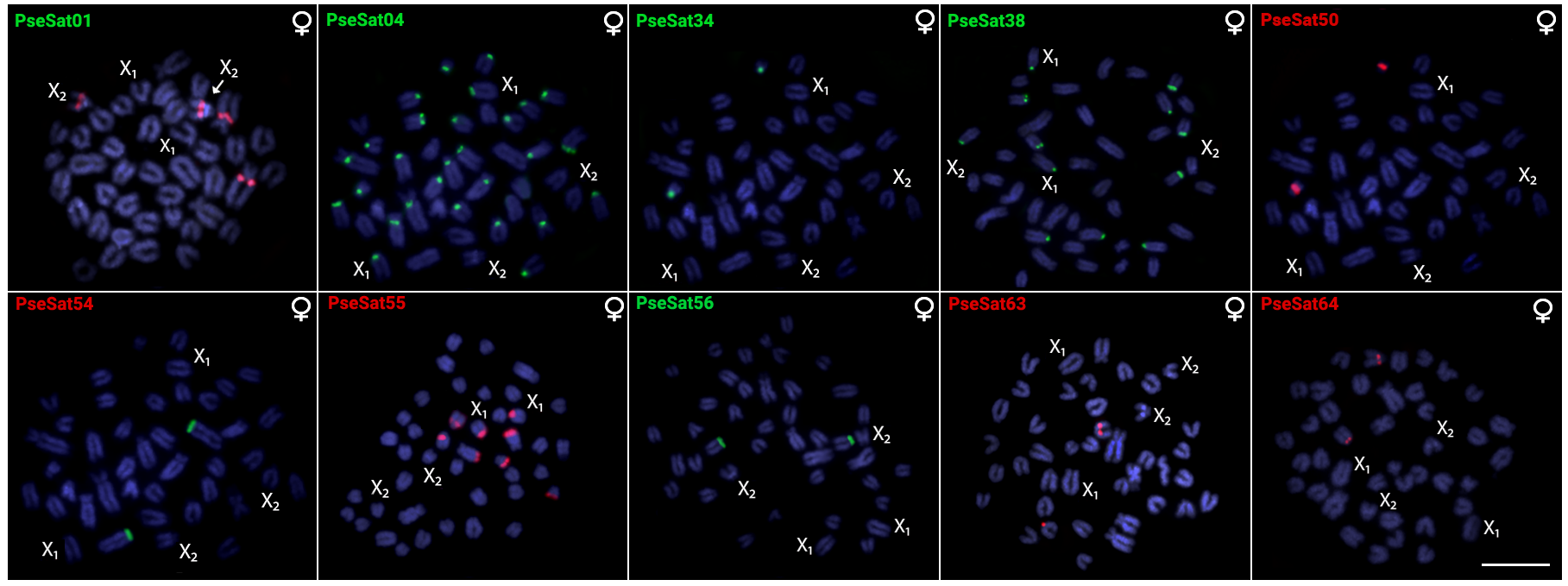

Supplement: Supplementary file 1 [file ijms-24-13654-s001.zip › Figure S1.tif]
